# Supplementary material for: The protein phosphatase 2A holoenzyme is a key regulator of starch metabolism and bradyzoite differentiation in Toxoplasma gondii
Source: Nat Commun. 2022 Dec 8;13:7560. doi: 10.1038/s41467-022-35267-5 (PMC9729606; doi:10.1038/s41467-022-35267-5)
Supplement: Supplementary file 1 — Supplementary Information File [file 41467_2022_35267_MOESM1_ESM.pdf]

Supplementary Information

The protein phosphatase 2A holoenzyme is a key regulator of starch metabolism and bradyzoite differentiation in *Toxoplasma gondii*

Jin-Lei Wang[1✉], Ting-Ting Li[1], Hany M. Elsheikha[2], Qin-Li Liang[1], Zhi-Wei Zhang[1], Meng Wang[1], L. David Sibley[3] & Xing-Quan Zhu[4✉]

[1]State Key Laboratory of Veterinary Etiological Biology, Key Laboratory of Veterinary Parasitology of Gansu Province, Lanzhou Veterinary Research Institute, Chinese Academy of Agricultural Sciences, Lanzhou, Gansu Province 730046, People's Republic of China

[2]Faculty of Medicine and Health Sciences, School of Veterinary Medicine and Science, University of Nottingham, Sutton Bonington Campus, Loughborough, LE12 5RD, UK

[3]Department of Molecular Microbiology, Washington University School of Medicine in St. Louis, St. Louis, MO 63110, USA

[4]Laboratory of Parasitic Diseases, College of Veterinary Medicine, Shanxi Agricultural University, Taigu, Shanxi Province 030801, People's Republic of China

✉emails: xingquanzhu1@hotmail.com (X.Q.Z.) and wangjinlei90@126.com (J.L.W.)

Supplementary Figures

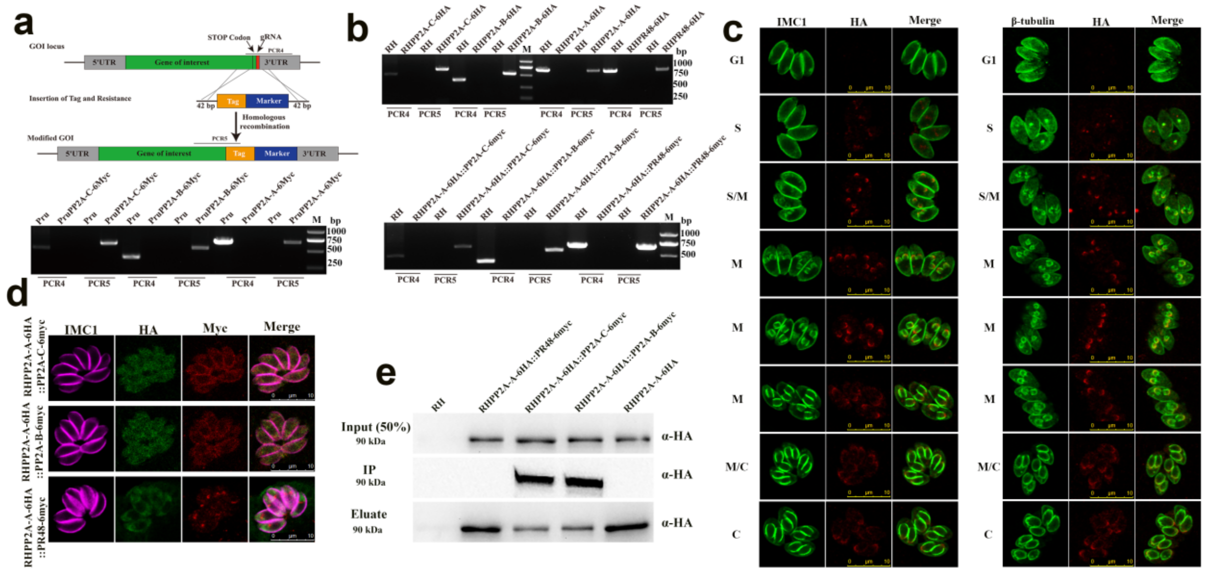

Supplementary Fig. 1. Identification of PP2A holoenzyme in *Toxoplasma gondii*. a Schematic representation of the gene of interest (GOI) endogenous tagging at the C-terminus. PCR4 produces a PCR product only in the wild-type strain with a short extension time (30 s) to identify whether the C-terminus was genetically modified. Successful insertion of the tag was detected by PCR5 and DNA sequencing. **b** PCR4 and PCR5 showed the correct integration of tags in the indicated strains, M: Marker. **c** Indirect immunofluorescence characterization of cell cycling of *T. gondii* RH tachyzoites expressing PR48-6HA under the control of an endogenous promoter and the tagged protein was detected using anti-HA antibody (red) and IMC1 and β-tubulin were stained green. Scale bar, 10 μm. **d** Co-localization of PP2A-A-6HA with PP2A-C-6Myc, PP2A-B-6Myc, and PR48-6Myc in RH strain, detected by indirect immunofluorescence with anti-HA (green), anti-Myc (red) and anti-IMC1(magenta) antibody. Scale bar, 10 μm. **e** Western blotting shows PP2A-A-6HA binding with PP2A-C-6Myc and PP2A-B-6Myc, while no binding was detected with PR48-6Myc. Samples were immunoprecipitated using anti-Myc antibody. Western blotting was performed on Myc-IP from RHPP2A-A-6HA::PP2A-C-6Myc, RHPP2A-A-6HA::PP2A-B-6Myc, and RHPP2A-A-6HA::PR48-6Myc, RHPP2A-A-6HA or whole cell lysate of RH using anti-HA antibody. Source data are provided as a Source Data file.

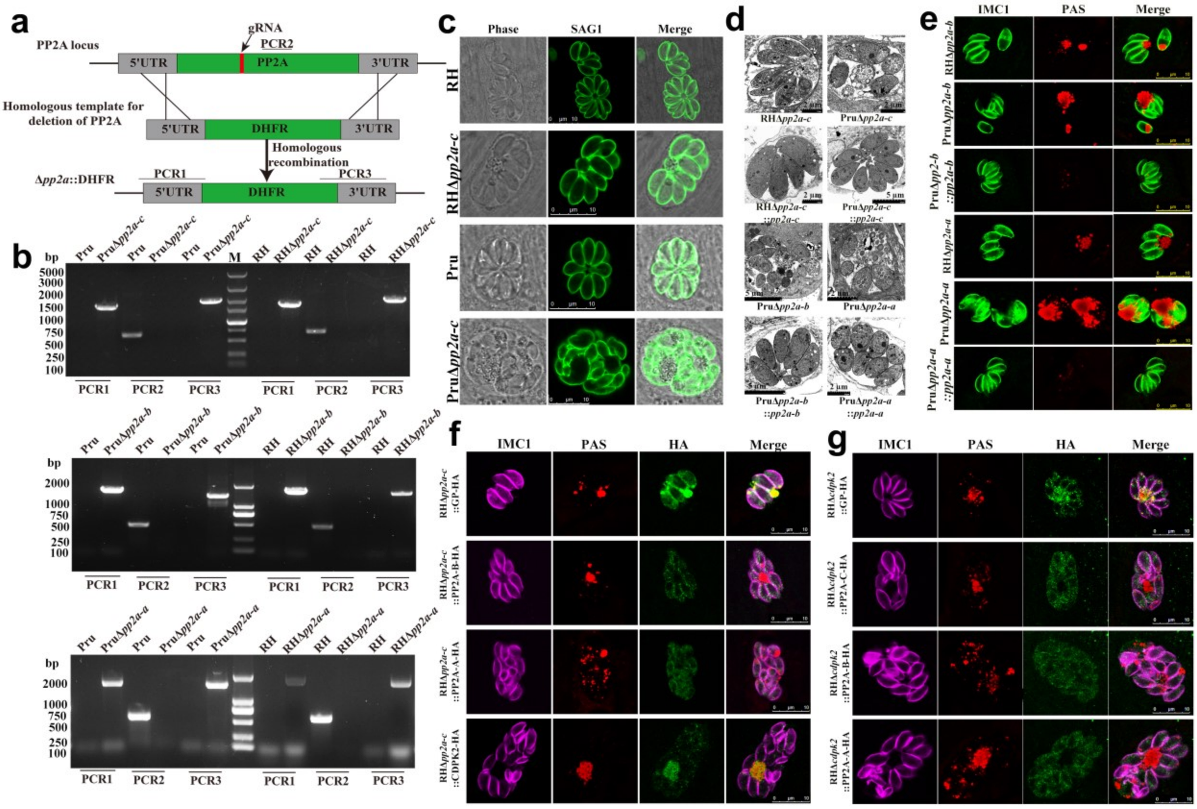

Supplementary Fig. 2. Disruption of PP2A holoenzyme causes aberrant amylopectin accumulation in *Toxoplasma gondii*. a Schematic representation of knocking out PP2A by CRISPR-Cas9-mediated homologous gene replacement in RH and Pru strains. PCR1 and PCR3 validated the 5′ and 3′ integration of the selection cassette, whereas PCR2 confirmed the successful deletion of the PP2A genes. **b** PCR confirmed the disruption of PP2A subunit. M: Marker. **c** Accumulation of granular deposits in RHΔ*pp2a-c* and PruΔ*pp2a-c* tachyzoites was visualized by IFA (staining with anti-SAG1) and bright-field microscopy. Scale bar, 10 μm. **d** Transmission electron microscopy micrographs showing granular deposits corresponding to semi-crystalline polysaccharide granules in Δ*pp2a-c*, Δ*pp2a-b*, Δ*pp2a-a* mutants, which were absent from the complemented strains. **e** Tachyzoites of the Δ*pp2a-a*, Δ*pp2a-b* mutant and the complemented strains were used to infect HFF cells under normal culture conditions and the amylopectin was stained with PAS (red), followed by immunofluorescence detection of tachyzoites with anti-IMC1 antibody (green). Scale bar, 10 μm. **f-g** PAS staining of the indicated parasites under normal culture conditions. The amylopectin was stained with PAS (red) and the indicated tagged protein was stained with anti-HA antibody, followed by immunofluorescence detection of parasites with anti-IMC1 antibody (magenta). Scale bar, 10 μm.

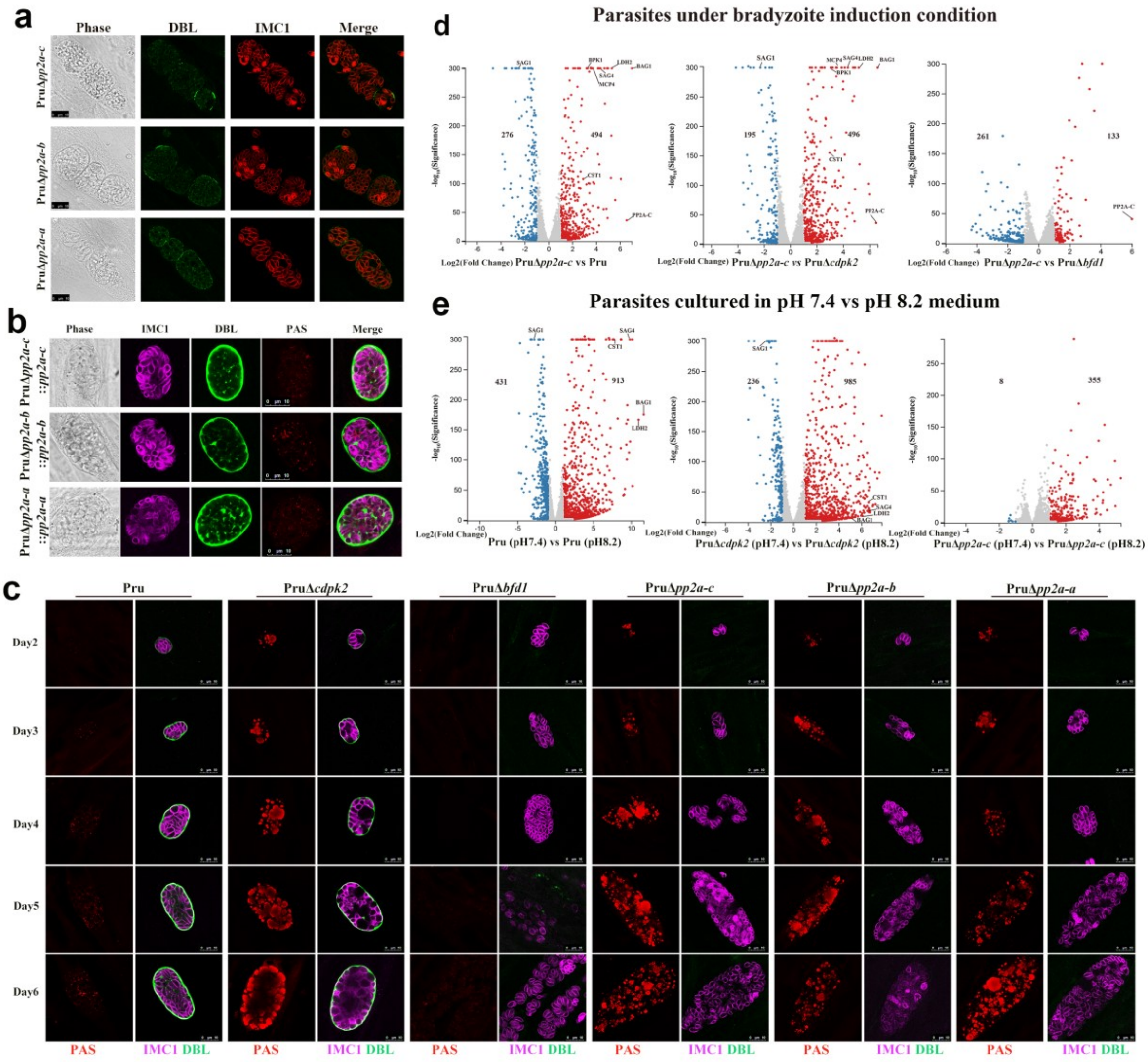

Supplementary Fig. 3. PP2A holoenzyme is necessary for stage differentiation in cell culture. **a** Representative vacuoles of the PP2A holoenzyme mutants were partly and weakly stained with FITC-*Dolichos biflorus* lectin (DBL) under alkaline culture conditions for 4 days. Scale bar, 10 µm. **b** The complemented PP2A holoenzyme knockout parasites restored the normal starch metabolism and bradyzoite differentiation. Scale bar, 10 µm. **c** PAS staining of the indicated parasites induced to differentiate into bradyzoites in alkaline medium over 6 days. The amylopectin was stained with PAS (red) and the bradyzoite cyst wall was stained with DBL (green). Immunofluorescence was used to detect the parasites with anti-IMC1 antibody (magenta). Scale bar, 10 µm. **d** Volcano plots show significant fold changes of the expressed genes of PruΔ*pp2a-c* vs Pru, PruΔ*pp2a-c* vs PruΔ*cdpk2* and PruΔ*pp2a-c* vs PruΔ*bfd1* under alkaline culture conditions. Genes with fold changes ≥ 2.0 or ≤ -2.0 with $P < 0.05$ are indicated in red and blue color, respectively. **e** Volcano plots show significant fold changes in genes from Pru, PruΔ*cdpk2*, and PruΔ*pp2a-c* strain under normal vs alkaline culture conditions. Genes with fold changes ≥ 2.0 or ≤ -2.0 with $P < 0.05$ are indicated in red and blue color, respectively.

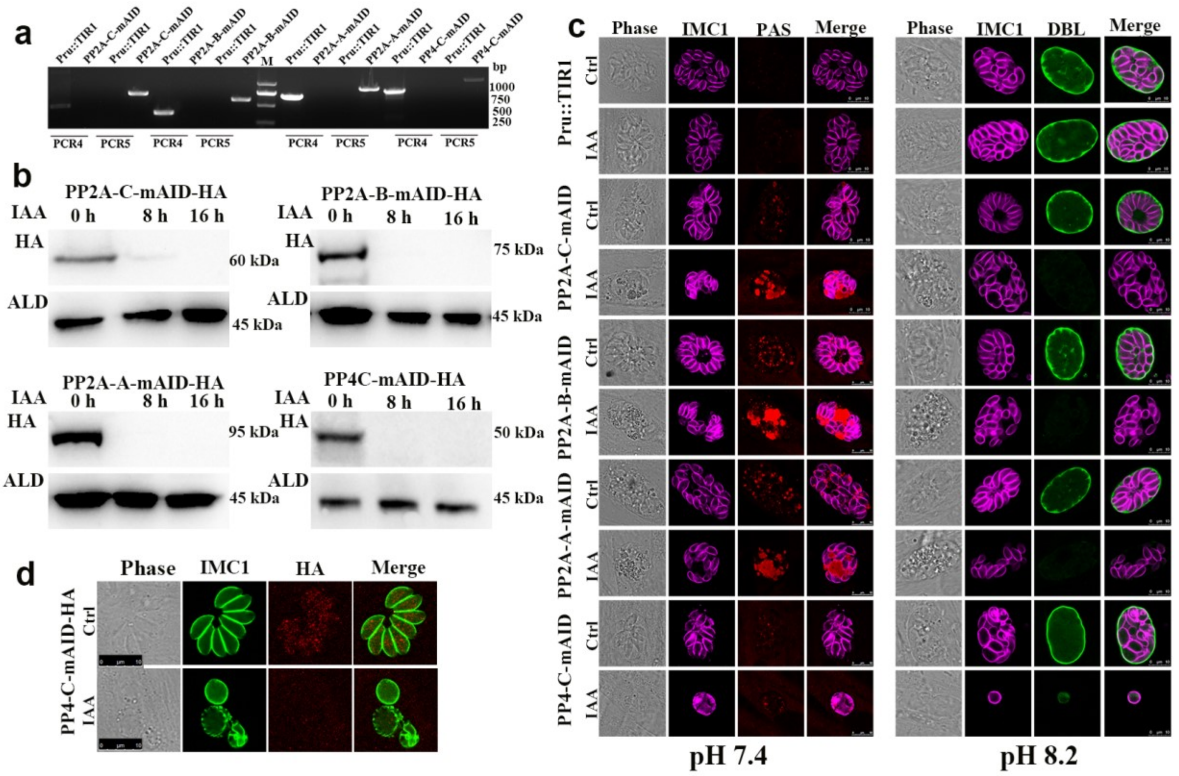

Supplementary Fig. 4. Conditional depletion of PP2A holoenzyme produces phenotype similar to that observed in PP2A mutant strains. a PCR confirmed the correct integration of mAID-HA tag in the indicated strains as described in Supplementary Fig 1a. M: Marker. **b** Western blotting of the total protein extract from the indicated strains treated with Auxin for different durations of time confirmed that the tagged protein is degraded by the mAID system. Western blots were probed with anti-HA to detect the presence of protein and ALD was used as a loading control. **c** Conditional depletion of PP2A with mAID system caused starch accumulation and blocked bradyzoite differentiation, while depletion of PP4C blocked parasite replication. The left panel shows mAID modified PP2A or PP4C strains infecting HFF cells under normal culture conditions and treated with IAA or vehicle for 36 h. The amylopectin was stained with PAS (red), followed by immunofluorescence detection of tachyzoites with anti-IMC1 antibody (magenta). The right panel shows mAID modified PP2A or PP4C strains infecting HFF cells for 4 h, followed by incubation in an alkaline culture medium treated with IAA or vehicle without CO_2 for 4 days for the induction of bradyzoites. Parasites were stained with anti-IMC1 antibody (magenta) and bradyzoite cyst wall was detected by FITC-*Dolichos biflorus* lectin (DBL) (green). Scale bar, 10 µm. **d** Immunostaining of the intracellular PP4C-mAID-HA parasites treated with IAA or vehicle for 24 h. Parasites were labeled with anti-IMC1 (green) and anti-HA (red) antibody. Scale bar, 10 µm. Source data are provided as a Source data file.

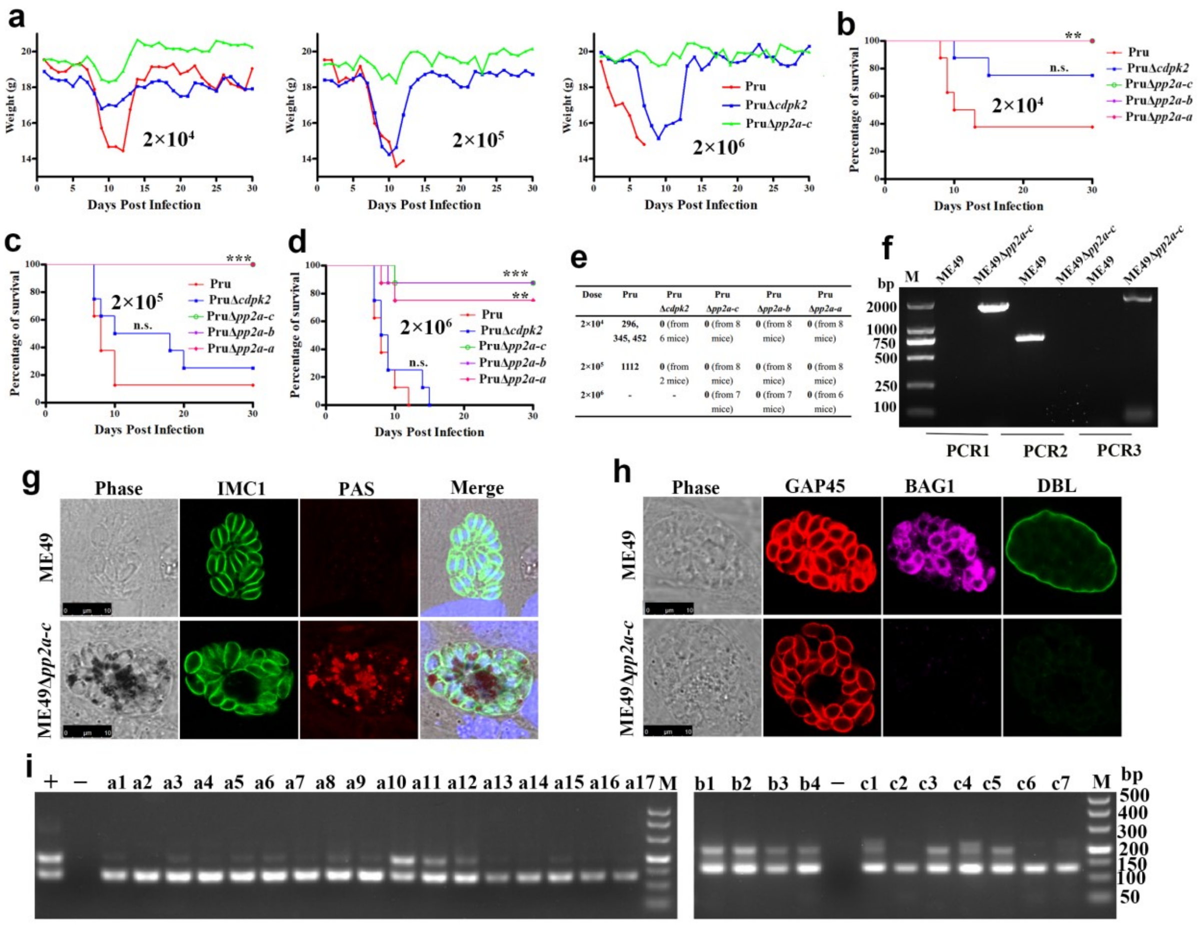

Dose	Pru	Pru Δcdpk2	Pru Δpp2a-c	Pru Δpp2a-b	Pru Δpp2a-a
2×10⁴	296, 345, 452	**0** (from 6 mice)	**0** (from 8 mice)	**0** (from 8 mice)	**0** (from 8 mice)
2×10⁵	1112	**0** (from 2 mice)	**0** (from 8 mice)	**0** (from 8 mice)	**0** (from 8 mice)
2×10⁶	-	-	**0** (from 7 mice)	**0** (from 7 mice)	**0** (from 6 mice)

Supplementary Fig. 5. PP2A holoenzyme mutants is important for virulence and brain cyst formation. a Average weights (grams) of C57BL/6 mice infected intraperitoneally with the indicated doses and strains. A weight of "0 g" indicates a mouse that was euthanized because they reached their humane endpoint. **b-d** Groups of eight Kunming female mice were infected intraperitoneally with the indicated doses, and the survival of mice was monitored for 30 days. Compared with Pru strain, for **b**, $^{**}P$ value = 0.0085 (Pru vs PruΔ*pp2a-c*), $^{**}P$ value = 0.0085 (Pru vs PruΔ*pp2a-b*), $^{**}P$ value = 0.0085 (Pru vs PruΔ*pp2a-a*), *P* value = 0.094 (Pru vs PruΔ*cdpk2*), n.s. = not significant; For **c**, $^{***}P$ value = 0.0005 (Pru vs PruΔ*pp2a-c*), $^{***}P$ value = 0.0005 (Pru vs PruΔ*pp2a-b*), $^{***}P$ value = 0.0005 (Pru vs PruΔ*pp2a-a*), *P* value = 0.3096 (Pru vs PruΔ*cdpk2*), n.s. = not significant; for **d**, $^{***}P$ value = 0.0001 (Pru vs PruΔ*pp2a-c*), $^{***}P$ value = 0.0002 (Pru vs PruΔ*pp2a-b*), $^{**}P$ value = 0.0011 (Pru vs PruΔ*pp2a-a*), *P* value = 0.3229 (Pru vs PruΔ*cdpk2*), n.s. = not significant; log rank Mantel-Cox test. **e** Brain tissue cyst burden of the survived mice from **b-d** was determined by FITC-*Dolichos biflorus* lectin (DBL) staining and was based on the number of cysts detected in one cerebral hemisphere per mouse. **f** PCR confirmed the disruption of PP2A-C subunit in ME49 strain. M: Marker. **g** Accumulation of granular deposits in ME49Δ*pp2a-c* tachyzoites. The wild-type ME49 and ME49Δ*pp2a-c* strains were inoculated into HFF cells under normal culture conditions for 36 h and the amylopectin was stained with PAS (red), followed by immunofluorescence detection of tachyzoites with anti-IMC1 antibody (green). Scale bar, 10 μm. **h** Disruption of PP2A-C in ME49 strain blocked parasite differentiation. The wild-type ME49 and ME49Δ*pp2a-c* strains were added into HFF cells for 4 h to allow the parasites to invade the host cells, followed by incubation in an alkaline culture medium without CO_2 for 4 days for the induction of bradyzoites. Parasites were stained with anti-GAP45 antibody (red) and the bradyzoite cyst was detected with DBL, while the bradyzoites were detected with specific anti-BAG1 antibody (magenta). **i** The results of PCR targeting *B1* gene showed the presence of *T. gondii* DNA in the brains of the survived mice. a1-a17 results from the survived mice infected with 10^2 ME49Δ*pp2a-c*; b1-b4 results from the survived mice infected with 10^2 ME49; c1-c7 from the survived mice infected with 10^5 ME49Δ*pp2a-c*. One sample from 10^5 ME49Δ*pp2a-c* group was not tested by PCR due to insufficiency of the brain tissue homogenate. +: Positive control; -: Negative control. Source data are provided as a Source data file.

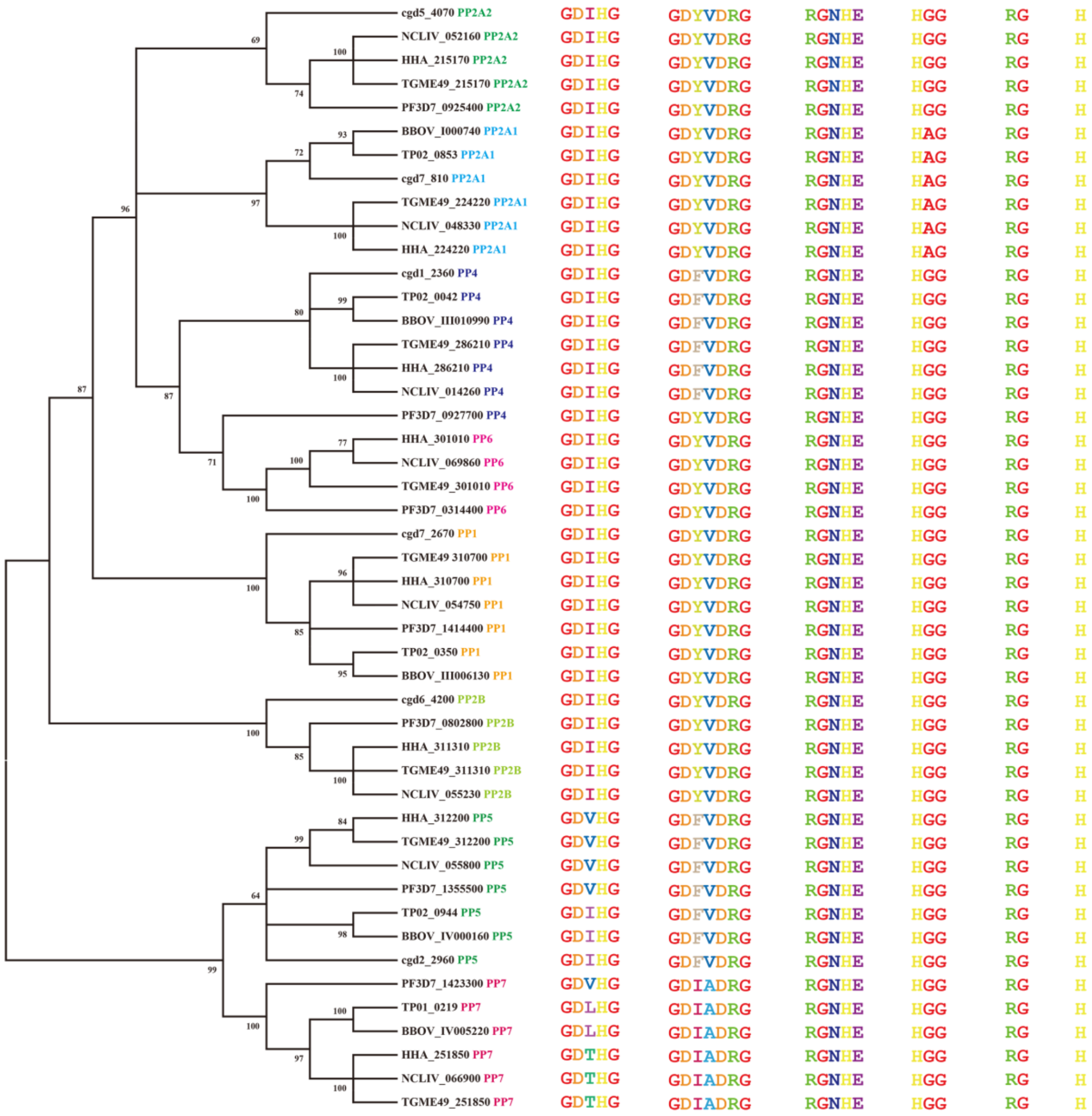

Supplementary Fig. 6. Phylogenetic analysis of phosphoprotein phosphatases in Apicomplexa. The phylogenetic tree was constructed based on the alignments of the catalytic domains of the PPP family phosphatase members (PP1, PP2A, PP2B, PP4, PP5, PP6 and PP7) found in the genomes of *Toxoplasma gondii* (TG), *Neospora caninum* (NC), *Hammondia hammondi* (HH), *Theileria parva* (TP), *Babesia bovis* (BB), *Plasmodium falciparum* (PF) and *Cryptosporidium parvum* (CP). Protein sequence alignment was performed by MUSCLE, and the conserved blocks selected by Gblocks 0.91b was used for tree-building. The phylogenetic analysis was performed by using MEGA 7.0 with a maximum likelihood method under the LG model of amino acid substitution. The final tree was condensed with a cut-off value 50%. The relative location and consensus sequences of each core catalytic motif are shown on the right. TgPP2A1 was denoted as PP2A in this study.

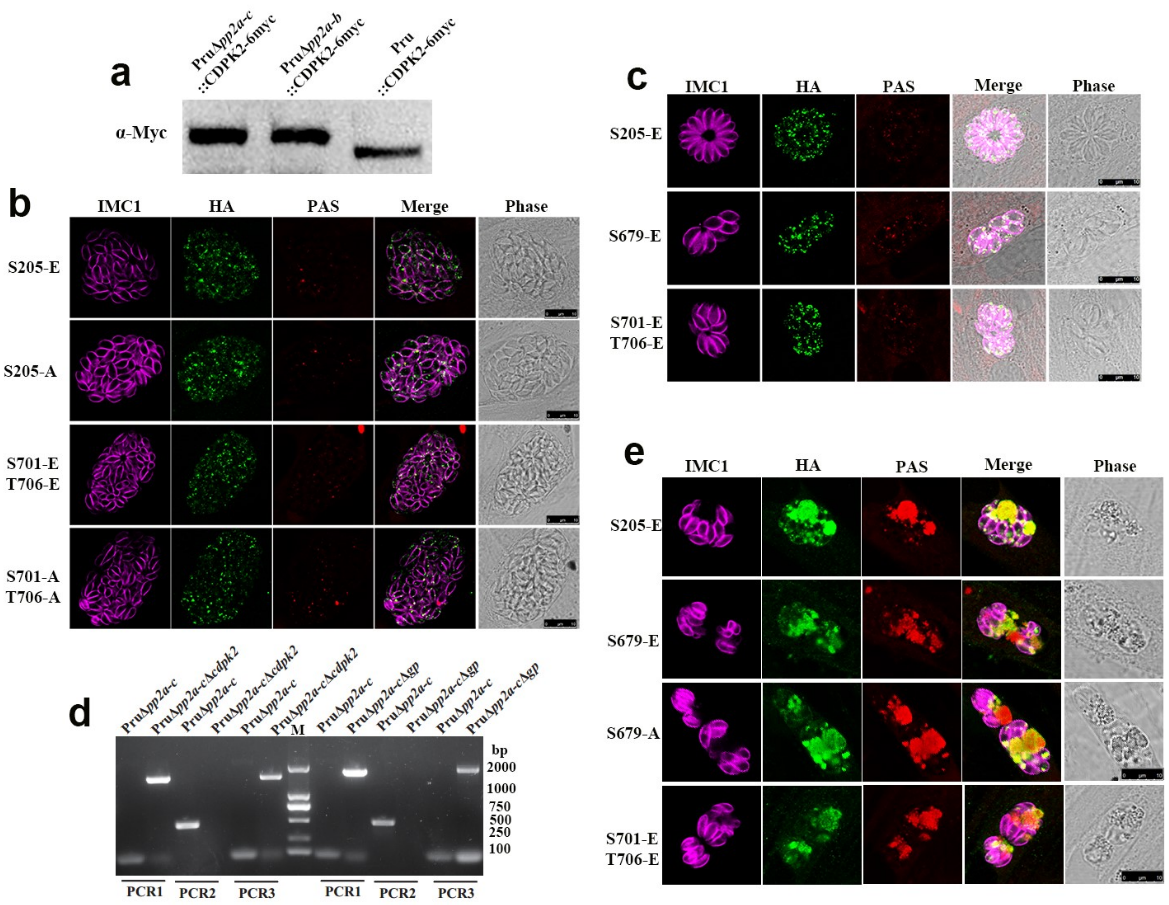

Supplementary Fig. 7. Other phosphomimetic CDPK2 mutations does not impair starch accumulation. a The migration of CDPK2 protein in the lysates from the endogenously tagged parental Pru, PruΔ*pp2a-c* or PruΔ*pp2a-b* strains was detected by Phos-tag gel electrophoresis. **b** PAS fluorescence of PruΔ*cdpk2* tachyzoites complemented with the other mutant version of CDPK2 fused with 3HA tags under the control of β-tubulin promoters and normal culture conditions. The amylopectin was stained with PAS (red) and the tagged protein was detected using α-HA antibody (green), and the parasites were detected with anti-IMC1 antibody (magenta). Scale bar, 10 μm. **c** PAS fluorescence of Pru tachyzoites expressing the phosphomimetic mutant version of CDPK2 fused with 3HA tags under the control of β-tubulin promoters and normal culture conditions. The amylopectin was stained with PAS (red) and the tagged protein was detected using α-HA antibody (green) and the parasites were detected with anti-IMC1 antibody (magenta). Scale bar, 10 μm. **d** PCR confirmed the disruption of CDPK2 or GP in PruΔ*pp2a-c* by CRISPR-Cas9 mediated homologous recombination as described in Supplementary Fig 2a. M: marker. **e** PAS fluorescence of PruΔ*pp2a-c*Δ*cdpk2* tachyzoites expressing the phosphomimetic mutant version of CDPK2 fused with 3HA tags under control of β-tubulin promoter and normal culture conditions. The amylopectin was stained with PAS (red) and the tagged protein was detected using α-HA antibody (green) and the parasites were detected with anti-IMC1 antibody (magenta). Scale bar, 10 μm. Source data are provided as a Source data file.

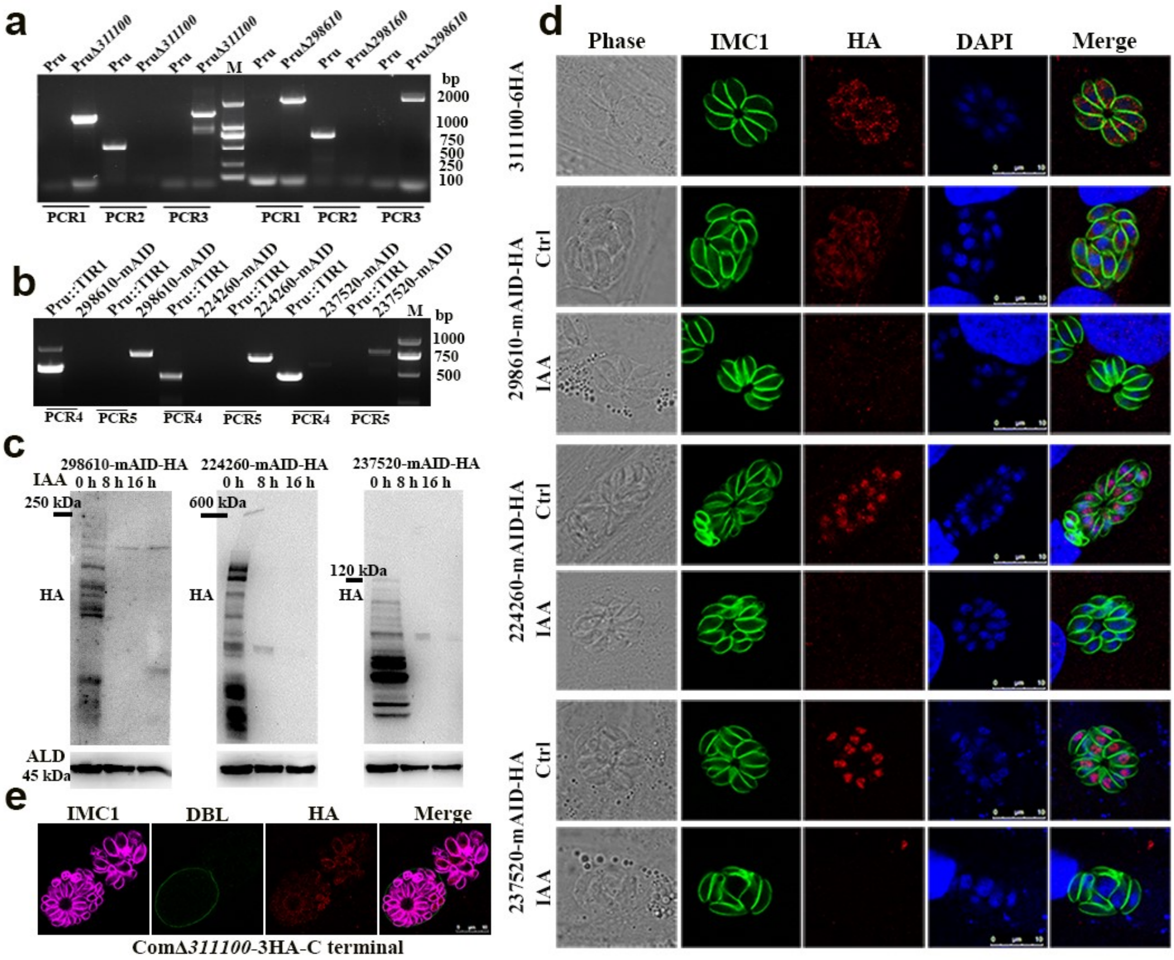

Supplementary Fig. 8. The four hyperphosphorylated proteins are important for parasite differentiation. a PCR confirmed the disruption of TGME49_311100 or TGME49_298610 in the Pru strain by CRISPR-Cas9 mediated homologous recombination as described in Supplementary Fig 2a. M: marker. **b** PCR confirmed the correct integration of tags in the indicated strains as described in Supplementary Fig 1a. M: marker. **c** Western blots were probed with anti-HA to detect mAID-HA tagged protein levels over a time course after addition of 500 µM IAA. The breakdown products of the tagged protein detected by immunoblotting may reflect instability in the parasite lysate or posttranscriptional modifications. ALD was used as a loading control. **d** A 6HA tag was fused to the C-terminus of the TGME49_311100 and a mAID-6HA tag was fused to the C-terminus of the TGME49_298610, TGME49_224260 and TGME49_237520. The mAID HA tagged protein was not detected after addition of IAA for 36 h. Scale bar, 10 µm. **e** Complementation with a 3HA tag fused to the C-terminus of TGME49_311100 in the Δ3*11100* strain did not restore bradyzoite differentiation. Scale bar, 10 µm. Source data are provided as a Source data file.
