# Supplementary material for: The protein phosphatase 2A holoenzyme is a key regulator of starch metabolism and bradyzoite differentiation in Toxoplasma gondii
Source: Nat Commun. 2022 Dec 8;13:7560. doi: 10.1038/s41467-022-35267-5 (PMC9729606; doi:10.1038/s41467-022-35267-5)
Supplement: Supplementary file 3 — Description of Additional Supplementary Information [file 41467_2022_35267_MOESM3_ESM.docx]

**Description of Additional Supplementary Files**

**File Name: Supplementary Data 1.** Summary of the mass spectrometry analysis of *Toxoplasma gondii* proteins immunoprecipitated with PP2A-B-6Myc or PP2A-A-6Myc.

**File Name: Supplementary Data 2.** Summary of the transcriptomic analysis of the parental Pru, PruΔ*cdpk2*, PruΔ*bfd1*, and PruΔ*pp2a-c* strains under normal culture conditions (pH 7.4) or alkaline medium (pH 8.2).

**File Name: Supplementary Data 3.** Summary of the proteomic analysis of the parental Pru, PruΔ*cdpk2*, and PruΔ*pp2a-c* strains under alkaline medium (pH 8.2).

**File Name: Supplementary Data 4.** Summary of the phosphoproteomic analysis of the parental Pru, PruΔ*cdpk2*, and PruΔ*pp2a-c* strains under alkaline medium (pH 8.2).

**File Name: Supplementary Data 5.** Quantitative data of *Toxoplasma gondii* phosphopeptides differentially phosphorylated (with a ≥ 2.0-fold difference) and the corresponding proteins between PruΔ*pp2a-c* vs Pru strains and PruΔ*pp2a-c* vs PruΔ*cdpk2* strains.

**File Name: Supplementary Data 6.** Primers and plasmids used in the study.
